# Targeting myeloid differentiation protein 2 by the new chalcone L2H21 protects LPS‐induced acute lung injury

**DOI:** 10.1111/jcmm.13017

**Published:** 2016-11-18

**Authors:** Yali Zhang, Tingting Xu, Beibei Wu, Hongjin Chen, Zheer Pan, Yi Huang, Liqin Mei, Yuanrong Dai, Xing Liu, Xiaoou Shan, Guang Liang

**Affiliations:** ^1^ Chemical Biology Research CenterSchool of Pharmaceutical SciencesWenzhou Medical UniversityWenzhouZhejiangChina; ^2^ The Second Affiliated HospitalWenzhou Medical UniversityWenzhouZhejiangChina; ^3^ Department of Orthopedic SurgeryThe First Affiliated HospitalWenzhou Medical UniversityWenzhouZhejiangChina; ^4^ Department of Oral Prophylaxis and HygieneSchool and Hospital of StomatologyWenzhou Medical UniversityWenzhouZhejiangChina

**Keywords:** acute lung injury, sepsis, myeloid differentiation 2, chalcone, LPS

## Abstract

Acute inflammatory diseases are the leading causes of mortality in intensive care units. Myeloid differentiation 2 (MD‐2) is required for recognizing lipopolysaccharide (LPS) by toll‐like receptor 4 (TLR4), and represents an attractive therapeutic target for LPS‐induced inflammatory diseases. In this study, we report a chalcone derivative, L2H21, as a new MD2 inhibitor, which could inhibit LPS‐induced inflammation both *in vitro* and *in vivo*. We identify that L2H21 as a direct inhibitor of MD‐2 by binding to Arg^90^ and Tyr^102^ residues in MD‐2 hydrophobic pocket using a series of biochemical experiments, including surface plasmon response, molecular docking and amino acid mutation. L2H21 dose dependently inhibited LPS‐induced inflammatory cytokine expression in primary macrophages. In mice with LPS intratracheal instillation, L2H21 significantly decreased LPS‐induced pulmonary oedema, pathological changes in lung tissue, protein concentration increase in bronchoalveolar lavage fluid, inflammatory cells infiltration and inflammatory gene expression, accompanied with the decrease in pulmonary TLR4/MD‐2 complex. Meanwhile, administration with L2H21 protects mice from LPS‐induced mortality at a degree of 100%. Taken together, this study identifies a new MD2 inhibitor L2H21 as a promising candidate for the treatment of acute lung injury (ALI) and sepsis, and validates that inhibition of MD‐2 is a potential therapeutic strategy for ALI.

## Introduction

Acute lung injury (ALI) and the acute respiratory distress syndrome are major causes of respiratory failure in critically ill patients 1. Sepsis, an acute inflammatory disease is the most common cause of ALI. Currently, sepsis is the 10th leading cause of death overall and accounts for major healthcare expenditures in the developing world 2. Despite extensive research, profound understanding of ALI pathogenesis and recent advances in supportive treatments, mortality rate from ALI remains high at approximately 40% 3. Effective pharmacotherapy for ALI and sepsis is extremely limited.

Research efforts in the field of sepsis and ALI have largely focused on the innate immune system and have conceptually viewed sepsis/ALI as a syndrome of hyperinflammation 4. Acute inflammation and cytokine storm have been implicated in the pathogenesis of ALI and sepsis. Lipopolysaccharide (LPS), known as an endotoxin, is a component in the outer membranes of Gram‐negative bacteria and is a potent initiator of local acute inflammation 5. The Toll‐like receptor (TLR) family members are key contributors to the endotoxin‐induced pro‐inflammatory conditions 6. Among TLRs, TLR4 was discovered to be a sensing receptor for bacterial LPS 7. Membrane‐bound TLR4 recognizes LPS and signals with enhanced efficiency after forming a receptor complex with accessory proteins, including myeloid differentiation protein 2 (MD‐2), LPS binding protein, and CD14 8, 9. As an obligate partner for TLR4, MD‐2 is required for localization of TLR4 on cell surface and for cell responsiveness to LPS 10, 11. In fact, LPS directly binds to a hydrophobic cavity in MD‐2, rather than TLR4, which induces MD‐2‐TLR4 combination and TLR4 activation, recruiting downstream MyD88 and triggering a pro‐inflammatory signal cascade 11. Activation of MD2/TLR4 by LPS leads to the overproduction of pro‐inflammatory cytokines, including interleukin (IL)‐6, tumour necrosis factor α (TNF‐α) and IL‐1β, which are demonstrated to be involved in the development and progression of ALI and sepsis.

**Figure 1:**
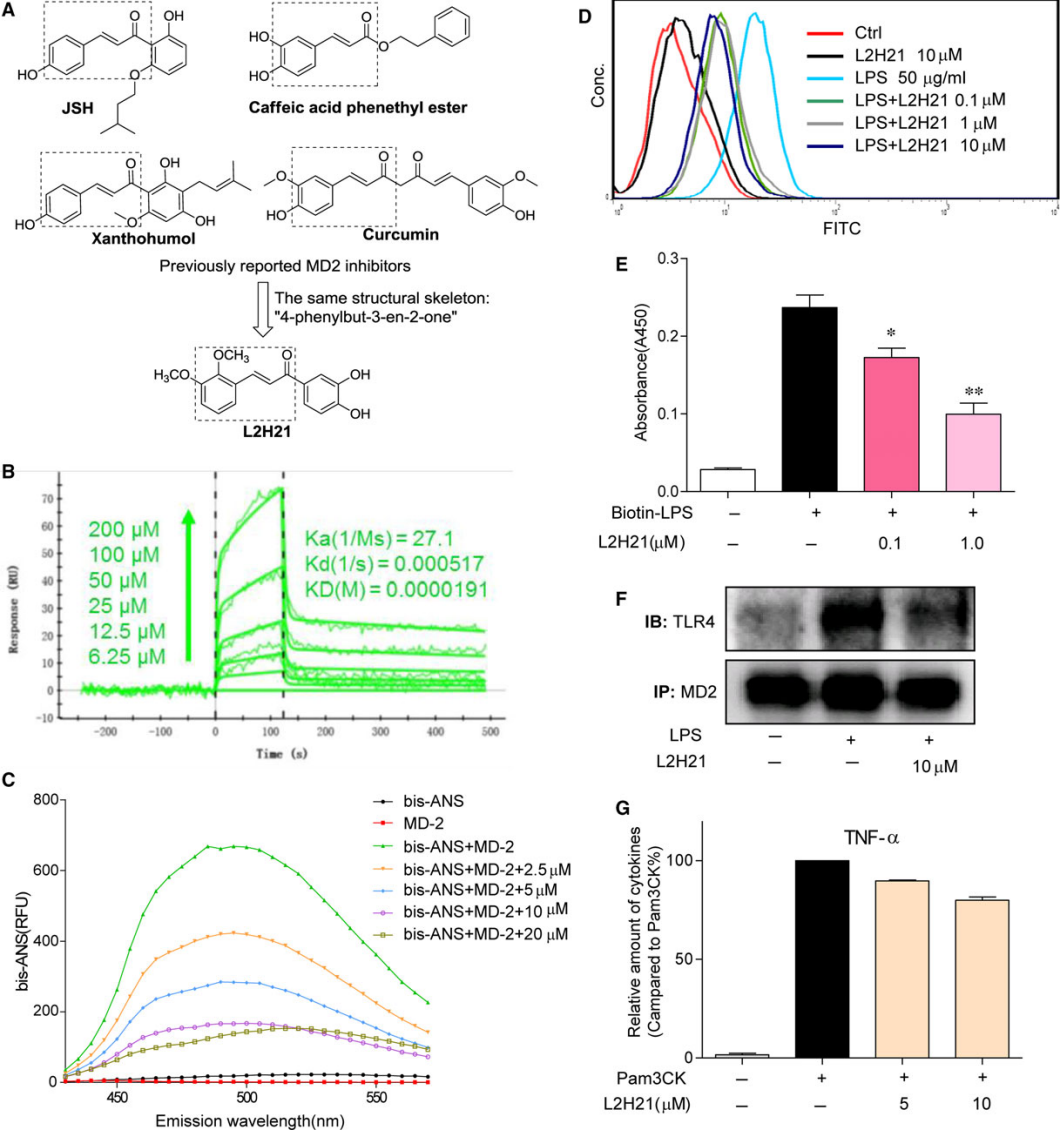
Antagonistic effect of L2H21 on LPS binding to rhMD‐2. (**A**) Chemical structures of current MD‐2 inhibitors and L2H21. (**B**) The binding affinity between L2H21 and rhMD‐2 was carried out by SPR. (**C**) After bis‐ANS (5 μM) pre‐incubated with rhMD‐2 (5 nM) to reach stable fluorescence values under excitation at 380 nm, then treated with L2H21 for 5 min. Emission spectra at 430–570 nm are represented as relative fluorescence units (RFUs). (**D**) HUVECs were incubated with DMSO alone (Ctrl), L2H21 (10 μM), FITC‐LPS (50 μg/ml) plus L2H21 (0.1 μM), FITC‐LPS (50 μg/ml) plus L2H21 (1 μM), FITC‐LPS (50 μg/ml) plus L2H21 (10 μM) or FITC‐LPS (50 μg/ml) alone for 30 min. These cells were collected and subjected to flow cytometry analysis. (**E**) The 96‐well plates for ELISA were coated with rhMD‐2 antibody at 4°C overnight. Then, rhMD‐2 protein was added to the plate and biotin‐LPS was added or not to the plate in the presence or absence of L2H21 (0.1 or 1 μM). LPS binding to rhMD‐2 was determined by ELISA, representing as absorbance values at 450 nm. Data are mean ± S.E.M. of three separate experiments performed in duplicate (*P < 0.05 and **P < 0.01 *versus* buffer‐alone‐added group). (**F**) MPMs (1 × 10^6^) were stimulated with LPS (1 μg/ml) for 5 min. in the presence or absence of L2H21 (10 μM). Cell extracts were subjected to the immunoprecipitation with anti‐MD‐2 antibody. Then the immune complexes were then immunoblotted with anti‐TLR4 and anti‐MD‐2 antibody. (**G**) RAW 264.7 macrophages (5 × 10^5^) were stimulated with Pam3CK (0.1 μg/ml) for 12 hrs in the presence or absence of L2H21 (5 and 10 μM), then the cytokine TNF‐ɑ level in the medium was detected by ELISA.

Pharmacological regulation of MD2/TLR4 activation can be beneficial for acute inflammatory diseases. As MD‐2 plays a critical role in LPS recognition, increasing studies indicate MD‐2 as a promising therapeutic target 12. Recent studies have demonstrated that MD‐2 is essential for intracellular distribution of TLR4 and TLR4's recognition of LPS 13. Previous reports found that MD‐2^−/−^ mice do not respond to LPS and do survive in endotoxin shock 14. Therefore, MD‐2 is proposed as a target for neutralizing the toxic effects of endotoxin. To date, several natural products have been found to exert anti‐inflammatory actions by directly binding and inhibiting MD2, such as JSH 15, caffeic acid phenethyl ester 16, xanthohumol 17 and curcumin 18 (Fig. 1A). Interestingly, the structures of these MD‐2 inhibitors share the same cinnamaldehyde skeleton. As part of long‐term efforts in natural medicines and novel anti‐inflammatories, our group synthesized and evaluated a series of chalcone derivatives as anti‐inflammatory agents on LPS‐stimulated macrophages. Among these synthetic chalcones, (*E*)‐2,3‐Dimethoxy‐3′,4′‐dihydroxychalcone (L2H21, Fig. 1A) showed excellent anti‐inflammatory activity. Due to similarity of chemical structure, we predicted MD‐2 as a target of L2H21. This study endeavours to identify the molecular target of L2H21 and to test its anti‐inflammatory effects in ALI and sepsis models. This study identifies L2H21 as a novel and specific MD‐2 inhibitor, which verifying the potential of L2H21 for development as an anti‐sepsis/ALI agent.

## Materials and methods

### Reagents

Compound L2H21 was provided by our laboratory and purified using HPLC with a purity of 99.3%. In *in vitro* experiments, L2H21 was dissolved in dimethyl sulphoxide (DMSO) solution and similar volume of DMSO followed as a vehicle control. In the *in vivo* mortality study, L2H21 was initially dissolved in water with macrogol 15 hydroxystearate (a non‐ionic solubilizer for injection from BASF) in water. The concentration of L2H21 and solubilizer was 2 mg/ml and 8% in water solution, respectively. For the vehicle, the solubilizer was prepared at 8% in water. In the *in vivo* ALI study, L2H21 was resuspended in 0.5% CMC‐Na solution. LPS, fluorescein isothiocyanate‐labelled LPS (FITC‐LPS) and Pam3CK were purchased from Sigma‐Aldrich (St. Louis, MO, USA). Anti‐CD68, anti‐MD‐2 and anti‐TLR4 antibodies were purchased from Santa Cruz (Santa Cruz, CA, USA). Recombinant human MD‐2 (rhMD‐2) protein was purchased from R&D Systems, Inc. (Minneapolis, MN, USA). Mutated rhMD‐2 protein was obtained by the methods described in our previous publication 19.

### Cell culture

Mouse RAW264.7 macrophages and human bronchial epithelial cell line (BEAS‐2B) were purchased from the Shanghai Institute of Biochemistry and Cell Biology (Shanghai, China). RAW264.7 macrophages were cultured in DMEM (Gibco, Eggenstein, Germany) containing 5.5 mM of D‐glucose supplemented with 10% foetal bovine serum (FBS; Gibco), 100 U/ml of penicillin (Gibco) and 100 mg/ml of streptomycin (Gibco). BEAS‐2B cells were cultured in RPMI‐1640 medium (Gibco) with the 20% FBS and antibiotic solution penicillin and streptomycin, at 37°C in a 5% CO_2_ atmosphere. Mouse peritoneal macrophages (MPMs) were obtained as previously described 20.

### Animals

Male C57BL/6 mice and ICR mice weighing between 18 and 22 g were obtained from the Animal Center of Wenzhou Medical University (Wenzhou, China). Animals were housed at a constant room temperature with a 12:12 hr light‐dark cycle and fed with a standard rodent diet for at least 7 days before used. All animal care and experimental procedures complied with the Wenzhou Medical University's Policy on the Care and Use of Laboratory Animals. Protocols for animal studies were approved by the Wenzhou Medical College Animal Policy and Welfare Committee (Approved documents: wydw2014‐0001).

### Docking of L2H21 to MD‐2

The molecular docking study was carried out with AutoDock version 4.2.6 21. The crystal structure of human MD‐2‐lipid IVa complex (PDB code 2E59) was obtained from Protein Data Bank for the docking simulation. The AutoDockTools version 1.5.6 package was applied to generate the docking input files and analyse the docking results. A 60 × 60 × 60 points grid box with a spacing of 0.375 Å between the grid points was implemented. The affinity maps of MD‐2 were calculated by AutoGrid. One hundred Lamarckian Genetic Algorithm runs with default parameter settings were processed. Then, we analysed the hydrogen bonds and bond lengths within the interactions of complex protein–ligand conformations.

### Fluorescence measurements of competition displacement

1,1′‐Bis(anilino)‐4,4′‐bis(naphthalene)‐8,8′‐disulfonate (bisANS, Carlsbad, CA, USA, 1 μM) and rhMD‐2 protein (5 nM) were mixed in PBS (pH 7.4) and incubated to reach stable fluorescence values under excitation at 385 nm. Non‐fluorescent L2H21 (2.5, 5, 10 or 20 μM) was then treated for 5 min., and followed by measuring relative fluorescence units emitted at 430–570 nm. Fluorescence measurements were performed with a SpectraMax M5 (Molecular Devices, CA, USA) at 25°C in a 1 cm path‐length quartz cuvette.

### ELISA binding of MD‐2 to LPS

ELISA for determination of L2H21's competition against LPS for binding to MD‐2 was performed in 96‐well plates. The 96‐well plates were coated with MD‐2 antibody at 4°C overnight and blocked with 3% bovine serum albumin (BSA) for 2 hrs at room temperature. Then, rhMD‐2, rhMD‐2/R90A or rhMD‐2/Y102A protein (4 μg/ml, respectively) diluted in 10 mM Tris‐HCl (pH 7.5) solution was added to the plate, incubated for 1.5 hrs and biotin‐LPS (InvivoGen, San Diego, CA, USA) was added to the plate in the presence or absence of L2H21 (0.1 or 1.0 μM). After incubated with horseradish peroxidase (HRP; Beyotime Biotech, Nantong, China) for 1 hr at room temperature, TMB (Beyotime Biotech) was added to the plate under dark condition for 15 min. The reaction was finally stopped with 2 N H_2_SO_4_ solution. The absorbance values were measured at 450 nm.

### Flow cytometric analysis

Cellular binding of FITC‐LPS was measured as described previously 22. Briefly, MPMs (1 × 10^6^) were incubated with FITC‐LPS (50 μg/ml) for 30 min. in the presence or absence of L2H21 (0.1, 1 or 10 μM). After washing, cells were collected and detected the amount of FITC‐LPS bound with cells were analysed by flow cytometry.

### Surface plasmon resonance analysis

The binding affinity of L2H21 to rhMD‐2 (rhMD‐2, or rhMD‐2 mutations) was determined with a ProteOn XPR36 Protein Interaction Assay system (Bio‐Rad Laboratories, Hercules, CA, USA) with an HTE sensor chip (ProteOn^™^, #176–5033). Briefly, rhMD‐2, rhMD‐2/R90A or rhMD‐2/Y102A (in acetate acid buffer pH 5.5) was loaded onto the sensors which were activated by 10 mM NiSO_4_. The L2H21 samples (at 200, 100, 50, 25, 12.5 and 6.25 μM) were prepared with a running buffer (PBS, 0.1% SDS, 5% DMSO). The sample plates were placed on the instrument. The interactions were determined according to the instructions of the manufacturer, at a flow rate of 30 ml/min. for 120 sec. during the association phase followed by 120 sec. for the dissociation phase at 25°C. The data were analysed with the ProteOn manager software. Equilibrium constant *K*_*D*_ values, the binding kinetic parameters, were calculated by a global fitting of the kinetic data from various concentrations of curcumin by a 1: 1 Langmuir binding model.

### Determination of TNF‐ɑ and IL‐6

The cytokine levels of TNF‐ɑ and IL‐6 in cell culture medium, bronchoalveolar lavage fluid (BALF) and serum from C57BL/6 mice for ALI study were determined with an ELISA kit (Bioscience, San Diego, CA, USA) according to the manufacturer's instructions. Mouse peritoneal macrophages and RAW 264.7 macrophages were seeded into 6‐well plates at a density of 40,000 cells per well. Cells were incubated at 37°C in 5% CO_2_ for 24 hrs. Cells were cultured with different concentrations (1, 2.5, 5 and 10 μM) of L2H21 or an equal volume of DMSO for 30 min., which was followed by the treatment of 0.5 μg/ml LPS. After treatment, the cells were incubated for another 24 hrs. The media were collected to measure the amount of TNF‐α and IL‐6. The total cytokines in the cell medium were normalized to the total protein amount of the viable cells. Experiments were performed at least three times *in vitro*.

### Co‐immunoprecipitation assay

Mouse peritoneal macrophages were cultured in 60‐mm plate and allowed to attach overnight at 37°C. Then, MPMs were pre‐treated with L2H21 (10 μM) or DMSO for 30 min. and then incubated with LPS (1 μg/ml) for 5 min. Total cells or lung tissues from ALI mice were lysed in an extraction buffer (containing cell protein extraction reagent supplemented with protease and phosphatase inhibitor cocktails) and centrifuged at 15616 × *g* for 10 min. at 4°C. Then, anti‐MD‐2 antibody (eBioScience, San Diego, CA, USA) was added into 400 μg proteins and gently rotated at 4°C overnight. The immunocomplex was collected with protein A+G agarose (Beyotime Biotech), and the precipitates were washed five times with ice‐cold PBS. Finally, proteins were released by boiling in sample buffer and utilized for Western blot analysis.

### Western blot assay

The protein samples from co‐immunoprecipitation were electrophoresed and then transferred to poly‐vinylidene difluoride transfer membranes. The blots were blocked for 2 hrs at room temperature with fresh 5% non‐fat milk in TBST and then incubated with anti‐MD‐2 or anti‐TLR4 antibody in TBST overnight at 4°C. Following three washes with TBST, the blots were incubated with HRP‐conjugated secondary antibodies for 1 hr, and the immunoreactivity bands were visualized with ECL kit (Bio‐Rad Laboratories).

### Real‐time quantitative PCR

Mouse peritoneal macrophages or BEAS‐2B cells were treated with 10 μM L2H21 for 30 min. and followed by 0.5 μg/ml LPS for 6 (MPMs) or 12 (BEAS‐2B) hours, respectively. Total RNAs were isolated from cells or lung tissues (10–20 mg) using the TRIzol method (Invitrogen, Carlsbad, CA, USA), and were then subjected to reverse transcription with a two‐step M‐MLV kit (Invitrogen). The Eppendorf Mastercycler ep realplex detection system (Eppendorf, Hamburg, Germany) was used for real‐time quantitative PCR (RT‐qPCR) analysis. The primers of genes including TNF‐ɑ, IL‐6, IL‐1β, COX‐2 and β‐actin were synthesized from Invitrogen (Shanghai, China). The amount of each gene was determined and normalized to the amount of β‐actin. The primer sequences used were listed as followed: mouse TNF‐ɑ sense, 5′‐TGATCCGCGACGTGGAA‐3′, mouse TNF‐ɑ antisense, 5′‐ACCGCCTGGAGTTCTGGAA‐3′; mouse IL‐6 sense, 5′‐GAGGATACCACTCCCAACAGACC‐3′, mouse IL‐6 antisense, 5′‐AAGTGCATCATCGTTGTTCATACA‐3′; mouse IL‐1β sense, 5′‐ACTCCTTAGTCCTCGGCCA‐3′, mouse IL‐1β antisense, 5′‐CCATCAGAGGCAAGGAGGAA‐3′; mouse COX‐2 sense, 5′‐TGGTGCCTGGTCTGATGATG‐3′, mouse COX‐2 antisense, 5′‐GTGGTAACCGCTCAGGTGTTG‐3′; mouse β‐actin sense, 5′‐CCGTGAAAAGATGACCCAGA‐3′, mouse β‐actin antisense, 5′‐TACGACCAGAGGCATACAG‐3′; human TNF‐ɑ sense, 5′‐CCCAGGGACCTCTCTCTAATC‐3′, human TNF‐ɑ antisense, 5′‐ATGGGCTACAGGCTTGTCACT‐3′; human IL‐6 sense, 5′‐GCACTGGCAGAAAACAACCT‐3′, human IL‐6 antisense, 5′‐TCAAACTCCAAAAGACCAGTGA‐3′; human IL‐1β sense, 5′‐ACGCTCCGGGACTCACAGCA‐3′, human IL‐1β antisense, 5′‐TGAGGCCCAAGGCCACAGGT‐3′; human COX‐2 sense, 5′‐TTCTCCTTGAAAGGACTTATGGGTAA‐3′, human COX‐2 antisense, 5′‐AGAACTTGCATTGATGGTGACTGTTT‐3′; human β‐actin sense, 5′‐CCTGGCACCCAGCACAAT‐3′, human β‐actin antisense, 5′‐GCCGATCCACACGGAGTACT‐3′.

### Animal experiment protocol of ALI

C57BL/6 mice received a continuously intragastric administration of L2H21 or 0.5% CMCNa (vehicle) and underwent intratracheal instillation of either LPS or 0.9% saline. Thus, four groups were created: sham mice given vehicle (Sham‐Vehicle group, *n* = 7), sham mice treated with L2H21 (Sham‐L2H21 group, *n* = 7), LPS mice given vehicle (LPS‐Vehicle group, *n* = 7) and LPS mice treated with L2H21 (LPS‐L2H21 group, *n* = 7). The animals were killed with an overdose of chloral hydrate at 6 hrs after intratracheal instillation LPS (5 mg/kg) or 0.9% saline. Then, the serum, BALF and lung tissues were collected.

### BALF analysis

Bronchoalveolar lavage fluid was performed through a tracheal cannula with 1.5‐ml saline solution by 3 times. After centrifuged at 243 × *g* for 5 min., the supernatant of BALF was immediately stored at −80°C before cytokines TNF‐α and IL‐6 analysis and the sediment was used for cell counting. Protein concentration of BALF was measured with the Bradford protein assay kit (Bio‐Rad Laboratories). The total number of cells in BALF was counted with a standard haemocytometer. Cell differentiation was examined by counting at least 200 cells with a light microscopy by cytospin and Wright‐Giemsa staining (Nanjing Institute of Biological Engineering, Nanjing, China).

### Lung Wet/Dry ratio

The middle lobe of right lung was collected, and the wet weight was recorded. Lung was then heated in a thermostatic oven at 65°C for 72 hrs and weighed to determine the baseline lung dry mass levels.

### Myeloperoxidase activity

To quantify neutrophil infiltration, Myeloperoxidase (MPO) activity in the homogenized lung tissues was determined with a MPO Detection Kit (Nanjing Jiancheng Bioengineering Institute, Nanjing, China). Lung tissue was homogenized in 1 ml of 50‐mM potassium PBS (pH 6.0) containing 0.5% hexadecyltrimethylammonium hydroxide and centrifuged at 15616 × *g* at 4°C for 20 min. Ten μl of the supernatant was transferred into PBS (pH 6.0) containing 0.17 mg/ml 3,3′‐dimethoxybenzidine and 0.0005% hydrogen peroxide. Myeloperoxidase activity of the supernatant was determined by using absorbance values measured *via* spectrophotometry at 460 nm and presented as units per gram of total protein (U/g). Total protein content in the samples was analysed using total protein assay.

### Lung histopathology

A portion of lung tissues were fixed in 4% paraformaldehyde solution and embedded in paraffin. The paraffin sections (5 μm) were stained with haematoxylin and eosin (Beyotime Biotech) to estimate the degree of lung injury by light microscopy (200× amplification; Nikon, Tokyo, Japan). Lung injury was graded from 0 (normal) to 4 (severe) in four categories: interstitial inflammation, neutrophil infiltration, congestion and oedema 23. Lung injury score was calculated by adding the individual scores for each category. Grading was performed by blinded pathologists. Lung injury score for each animal was calculated as the mean of four lung sections. Paraffin sections were obtained from each mouse at 6 hrs after intratracheal instillation.

### Immunofluorescence for CD68 detection

After deparaffinization and rehydration, 5‐μm lung tissue sections on the slides were incubated with 1% BSA in PBS for 30 min. Slides were incubated overnight at 4°C with anti‐CD68 antibody (1:100), then followed by 1 hr incubation with a FITC‐labelled secondary antibody (Santa Cruz) at 37°C. The nucleus was stained with 4′,6‐diamidino‐2‐phenylindole (Beyotime Biotech) for 5 min. in room temperature. The images were viewed under a fluorescent microscope (400× amplification; Nikon).

### LPS‐induced inflammatory mortality in mice

Compound was firstly dissolved with macrogol 15 hydroxystearate (a non‐ionic solubilizer for injection from BASF) with or without medium‐chain triglycerides (MCT, from BASF) in water bath at 37°C. The concentration of compounds was 2 mg/ml. The concentration of solubilizer was ranged 5–10%, and MCT 0.5–2% in final solution. For the vehicle, the mixture of solubilizer and MCT was prepared at 10% and 2%, respectively. Male C57BL/6 mice weighing 18–22 g were pre‐treated with L2H21 (20 mg/kg) in a water solution by intraperitoneal (*i.p*.) injection 15 min. before or after the intravenous (*i.v*.) injection of LPS (20 mg/kg). Control animals received a similar volume (200 μl) of vehicle. Body weight changes and mortality rate were recorded for 7 days.

### Statistical analysis

The results were shown as means ± S.E.M. The statistical significance of differences between two groups was analysed by means of Student's *t*‐test. *P* < 0.05 was considered statistical significance. All the experiments related to cells were repeated at least three times.

## Results

### L2H21 is a directly inhibitor of MD‐2

We firstly determined the direct interaction between L2H21 and rhMD‐2 protein with surface plasmon resonance (SPR) analysis and fluorescence spectroscopy. The SPR experiments indicate that L2H21 directly binds MD‐2 protein in a dose‐dependent manner and with a very high affinity (K_D_ value = 19.1 μM) (Fig. 1B), higher than that of L6H21, a previously reported MD‐2 inhibitor sharing the chalcone structure (K_D_ value = 33.3 μM) 24. Next, bis‐ANS, a fluorescent probe to map the lipid‐binding sites in protein, was used to test MD‐2–L2H21 interaction. Fluorescence values of bis‐ANS were markedly enhanced upon binding to rhMD‐2 protein, while incubation with 2.5, 5, 10 and 20 μM L2H21 dose dependently decreased the fluorescence value of bis‐ANS, suggesting that L2H21 competitively binds to the hydrophobic pocket of rhMD‐2 protein (Fig. 1C). The above data indicate a direct interaction between L2H21 and MD‐2 protein.

To investigate whether L2H21 effectively inhibits LPS–MD‐2 interaction at the cell level, MPMs were incubated with FITC‐LPS either with or without L2H21, and then subjected to flow cytometry analysis. As shown in Figure 1D, treatment with L2H21 reduced the binding of FITC‐LPS to the cell surface membrane in a dose‐dependent manner. In addition, a cell‐free ELISA analysis, using biotin‐labelled LPS, showed that the biotin‐LPS strongly binds to rhMD‐2, while L2H21 significantly inhibits binding of biotin‐LPS to rhMD‐2 (Fig. 1E). Upon LPS stimulation, TLR4 and MD‐2 form a complex to activate the signalling pathway; thus, we examined the effect of L2H21 on LPS‐induced TLR4/MD‐2 complex formation. As shown in Figure 1F, pre‐treatment with 10‐μM L2H21 for 30 min. markedly blocked the LPS‐induced formation of TLR4/MD‐2 complex in macrophages.

Next, the targeting specificity of L2H21 on MD‐2 was tested. As MD‐2 is not required to activate the TLR2 pro‐inflammatory signalling pathway, Pam3CK, an agonist of TLR2, served to stimulate an inflammatory response in MPMs. Mouse peritoneal macrophages were pre‐treated with 5 or 10 μM L2H21 for 30 min. followed by Pam3CK stimulated cells for 12 hrs. The result indicates that Pam3CK significantly induced the TNF‐α production and L2H21 failed to suppress TLR2‐mediated TNF‐α release in macrophages (Fig. 1G), in turn which indicates that the anti‐inflammatory effects of L2H21 are MD‐2 dependent.

### Study on the binding mechanism between L2H21 and MD‐2

**Figure 2:**
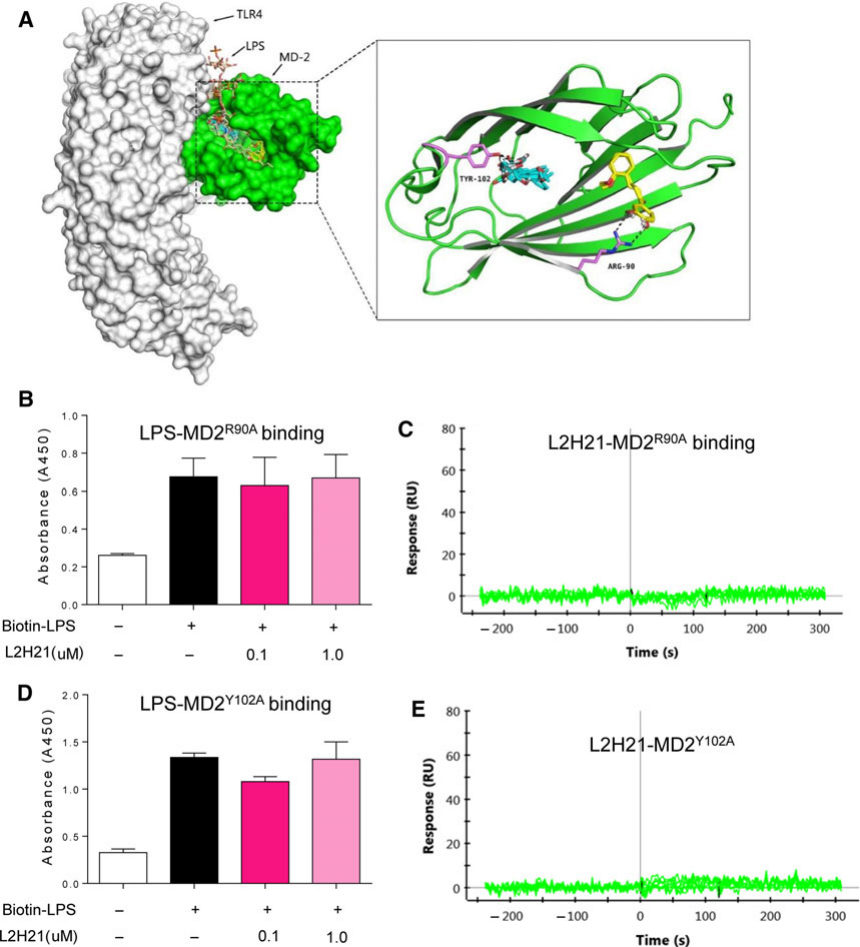
Antagonistic mechanism of L2H21 on LPS binding to MD‐2. (**A**) Molecular docking of L2H21 with MD‐2 protein was carried out with the programmer Tripos molecular modelling packages Sybyl‐x.v1.1.083. (**B** and **D**) The 96‐well plates for ELISA were coated with rhMD‐2 antibody at 4°C overnight. Then, rhMD‐2^R90A^ or rhMD‐2^Y102A^ protein was added to the plate and biotin‐LPS was added or not to the plate in the presence or absence of L2H21 (0.1 or 1 μM). LPS binding to rhMD‐2 was determined by ELISA, representing as absorbance values at 450 nm. Data are mean ± S.E.M. of three separate experiments performed in duplicate. (**C** and **E**) The binding affinity between L2H21 and rhMD‐2^R90A^ or rhMD‐2^Y102A^ was carried out by SPR.

To investigate the underlying structural mechanism of the L2H21 binding to MD‐2 protein, docking software simulated the molecular L2H21–MD‐2 complex. As shown in Figure 2A, the whole L2H21 molecule lodges inside the hydrophobic pocket of MD‐2, overlapping a portion of the LPS binding sites. Computer‐assisted prediction shows that L2H21 fits the hydrophobic pocket of MD‐2, interacting with the residues, including Tyr^102^ and Arg^90^, in the most energy‐favourable configuration. To better understand the role of Arg^90^ and Tyr^102^ in the L2H21 binding to MD‐2, two MD‐2 point mutation proteins (R90A mutation and Y102A mutation) were constructed. Results from Figure 2B and D indicate that L2H21 at 0.1 or 1.0 μM fails to inhibit binding between biotin‐LPS and MD‐2^R90A^ or MD‐2^Y102A^ using the assay of MD2‐cell‐free ELISA. Different concentrations (200, 100, 50, 25, 12.5 and 6.25 μM) of L2H21 flow past the chip fixed with MD‐2^R90A^ or MD‐2^Y102A^ exhibit no signalling response change using the SPR assay (Fig. 2C and E). These results indicate that L2H21 no longer binds to the two named mutants. Together, the above results demonstrate a possible binding mechanism between L2H21 and MD‐2 protein.

### L2H21 inhibits LPS‐stimulated inflammatory cytokine production

**Figure 3:**
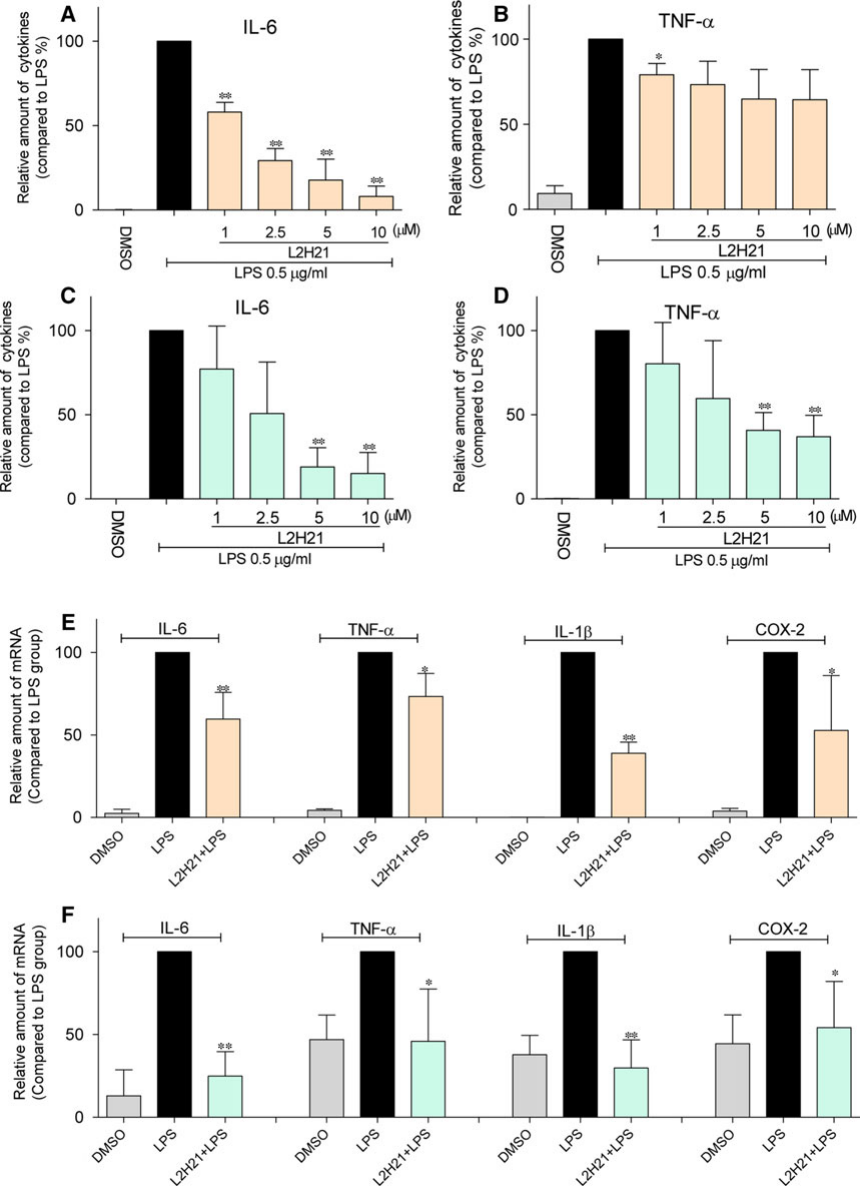
L2H21 inhibited the LPS‐induced inflammatory response *in vitro*. RAW 264.7 macrophages (**A** and **B**) or MPMs (**C** and **D**) were incubated with LPS (0.5 μg/ml) in the presence or absence of L2H21 (1, 2.5, 5 or 10 μM) for 24 hrs. The inflammatory cytokines levels of TNF‐α and IL‐6 in medium were measured by ELISA. MPMs (**E**) or Beas‐2B cells (**F**) were incubated with LPS (0.5 μg/ml) in the presence or absence of L2H21 (10 μM) for 6 hrs. Total mRNA was extracted from the cells using TRIzol, and the mRNA levels of TNF‐α, IL‐6, IL‐1β and COX‐2 were detected by real‐time qPCR analysis. Data are mean values (±S.E.M.) of at least three separate experiments. Statistical significance relative to LPS group was indicated, *P < 0.05, **P < 0.01.

After being pre‐treated with different concentrations (1, 2.5, 5 and 10 μM) of L2H21, mouse RAW 264.7 macrophages (Fig. 3A and B) or MPMs (Fig. 3C and D) were stimulated with LPS for 24 hrs. Presented in Figure 3A and B, testing reveals that pre‐treatment with L2H21 dose dependently decreases the LPS‐induced production of IL‐6 and TNF‐α. Similar results were observed in MPMs (Fig. 3C and D). Moreover, pre‐treatment of MPMs with 10‐μM L2H21 followed with LPS‐stimulated cells for 6 hrs markedly attenuates LPS‐induced mRNA levels of TNF‐α, IL‐6, IL‐1β and COX‐2 in MPMs (Fig. 3E). Furthermore, similar results were observed in human airway epithelial (BEAS‐2B) cells, where 10‐μM L2H21 significantly suppressed LPS‐induced TNF‐α, IL‐6, IL‐1β and COX‐2 transcription (Fig. 3F).

### L2H21 protects ALI in mice by targeting MD‐2

**Figure 4:**
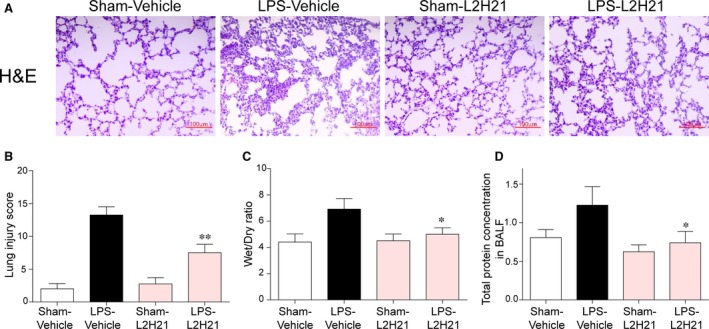
Effects of L2H21 on LPS‐induced ALI. L2H21 (20 mg/kg) was consecutively administrated to C57BL/6 mice for 7 days by intragastric administration, and 6 hrs after intratracheal stimulation of LPS (5 mg/kg), the right lung and trachea was isolated. (**A**) The lung histological changes were assessed by haematoxylin and eosin staining. (**B**) The histogram of lung injury scores based on A. (**C**) The lung Wet/Dry weight ratio. (**D**) Protein concentration in BALF. The values presented are the means ± S.E.M. *P < 0.05 and **P < 0.01 *versus* LPS group.

C57BL/6 mice in Sham‐L2H21 and LPS‐L2H21 groups were treated with 20 mg/kg L2H21 by daily gavage for 7 days and then LPS‐Vehicle and LPS‐L2H21 groups were given 5 mg/kg LPS. As shown in Figure 4A, lung architecture of ALI mice indicates infiltration of inflammatory cells in lung interstitium and alveolar spaces as well as alveolar wall thickening and congestion, whereas pre‐treatment with L2H21 attenuates these LPS‐induced changes. Here, the lung injury scores were semi‐quantitatively assessed by a blinded pathologist (Fig. 4B). Lung wet/dry weight ratio is an index of lung oedema. We assessed pulmonary oedema caused by LPS instillation (Fig. 4C) through lung wet/dry ratio, and protein concentrations in BALF (Fig. 4D), which is a feature of alveolar–capillary barrier damage. Administration of L2H21 clearly reduces the LPS‐induced pulmonary oedema and the increase in protein concentrations in BALF.

**Figure 5:**
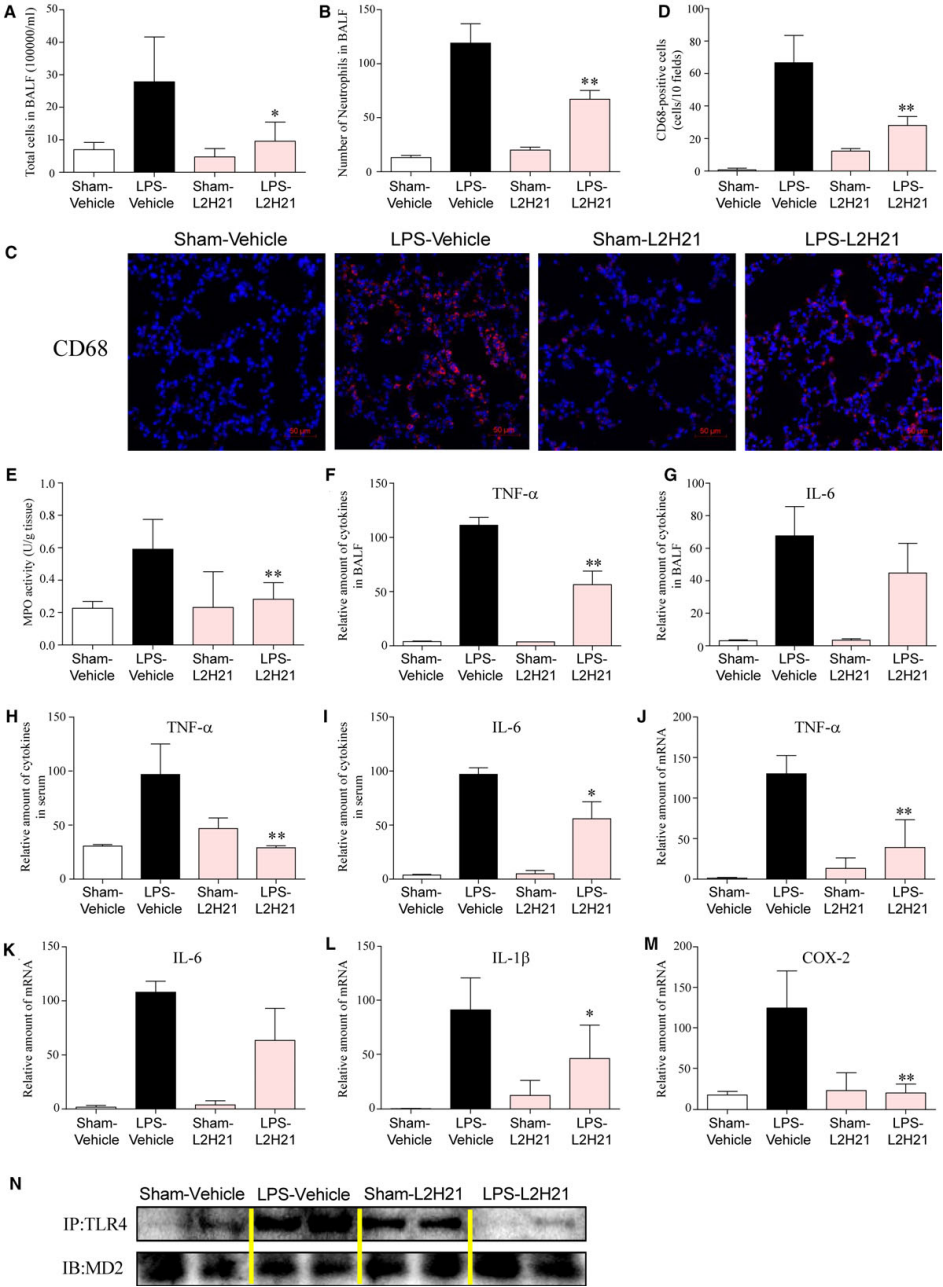
L2H21 attenuated the LPS‐induced inflammatory response by targeting MD‐2 in lung tissue. The effects of L2H21 on total inflammatory cells (**A**) and neutrophils (**B**) infiltration in BALF. (**C**) Macrophages infiltration was determined by CD68 immunofluorescence staining. (**D**) The number of CD68‐positive cells in 10 fields based on C. (**E**) MPO activity in lung tissue. (**F**) The effect of L2H21 on the amount of TNF‐α in BALF. (**G**) The effect of L2H21 on the amount of IL‐6 in BALF. (**H**) The effect of L2H21 on the amount of TNF‐α in serum. (**I**) The effect of L2H21 on the amount of IL‐6 in serum. (**J**–**M**) The mRNA level of TNF‐α (**J**), IL‐6 (**K**), IL‐1β (**L**) and COX‐2 (**M**) in lung tissue was determined by RT‐qPCR. (**N**) L2H21 inhibited the formation of MD‐2/TLR4 complex in lung tissue. The values presented are the means ± SEM. *P < 0.05 and **P < 0.01 *versus* LPS group.

Next, we evaluated the effect of L2H21 on LPS‐induced inflammation in the lung tissue. Exposure to LPS caused an increase influx of total cells (Fig. 5A) and neutrophils (Fig. 5B) into BALF, whereas administration of L2H21 reduced cells infiltration. Macrophages infiltration of ALI mice lung tissue was detected by CD68 immunofluorescence staining. Treatment by L2H21 significantly inhibits infiltration by macrophages (Fig. 5C), which can be intuitively observed by CD68‐positive cells counts in 10 fields (Fig. 5D). Increased MPO activity reflects increased neutrophil infiltration of lung tissue. As shown in Figure 5E, L2H21 attenuates the increased MPO activity as induced by LPS in lung tissue. Meanwhile, L2H21 also inhibits the LPS‐induced increase in TNF‐α and IL‐6 both in BALF (Fig. 5F and G) and in serum (Fig. 5H and I), although the inhibitory effects of L2H21 on IL‐6 levels in BALF do not have significant differences. The transcriptional levels of inflammatory cytokines of TNF‐α (Fig. 5J), IL‐6 (Fig. 5K), IL‐1β (Fig. 5L) and COX‐2 (Fig. 5M) induced by ALI in lung tissue were also reduced by pre‐treatment with 20 mg/kg L2H21. For confirmation that L2H21 is an inhibitor of MD‐2 *in vitro*, the level of TLR4/MD‐2 complex in lung tissues was determined by immunoprecipitation assay. As shown in Figure 5N, L2H21 markedly reduced LPS‐induced formation of TLR4/MD‐2. These data illustrates that L2H21 exhibits protection against ALI by targeting MD‐2 *in vivo*.

### L2H21 effectively protects mice from LPS‐induced mortality

**Figure 6:**
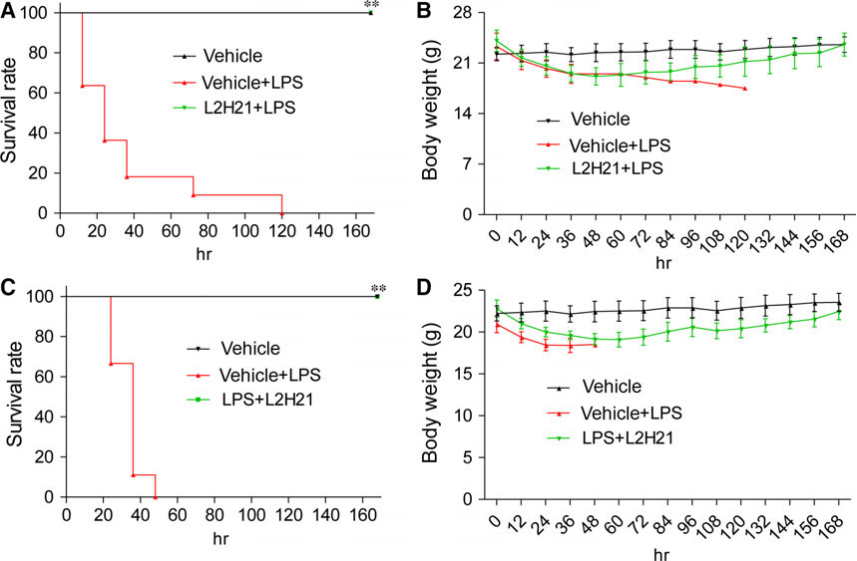
L2H21 attenuated LPS‐induced septic shock *in vivo*. Male C57BL/6 mice were treated with L2H21 (*i.v*., 20 mg/kg) 15 min. before (**A** and **B**) of after (**C** and **D**) injection of LPS (*i.v*., 20 mg/kg). Survival (**A** and **C**) and body weight (**B** and **D**) were recorded for 7 days at an interval of 24 hrs after the LPS injection. **P < 0.01 *versus* LPS group.

Mice weighing 18–22 g were injected with 20 mg/kg LPS in the caudal vein either with or without L2H21 at a dose of 20 mg/kg for *i.v*. administration. Survival rates and body weight of mice were monitored for 7 days. As illustrated in Figure 6A and C, all animals treated with LPS alone (LPS group) died within 5 days and more than 80% of mice died within 2 days as a result of septic shock. However, survival rates reached 100% in animals that received L2H21 15 min. before (for prevention) (Fig. 6A) or 15 min. after (for therapy) (Fig. 6C) LPS injection. Simultaneously, mice in both prevention group and treatment groups lost weight the first 0–2 days after the LPS procedure, but regained normal weight gradually over the following 5 days (Fig. 6B and D). Thus, results here demonstrate the anti‐inflammatory effects of MD‐2 inhibitor, as well as L2H21's potent prevention and treatment effects in sepsis.

## Discussion

In this study, we identified L2H21 as an inhibitor of MD‐2 by the mechanism of binding to residues of Arg^90^ and Tyr^102^ in the MD‐2 hydrogen pocket. In addition, L2H21 displays a remarkable alleviation effect on inflammatory response as induced by LPS in macrophages and human airway epithelial cells. *In vivo*, L2H21 can also attenuate LPS‐induced ALI by targeting MD‐2. As well, L2H21 exhibits protective effects in mice from LPS‐induced mortality.

Myeloid differentiation 2, a co‐receptor for TLR4, physically associates with TLR4 on the cell surface and mediates the interaction between LPS and TLR4 11. Myeloid differentiation 2 knockout mice showed no response to LPS and LPS‐induced inflammation 14. Due to its essential role in activating the TLR4 signalling pathway of bacterial LPS, MD‐2 represents a more attractive target than TLR4 for inhibition of LPS‐induced inflammatory disorders. In recent years, MD‐2 inhibition by pharmacological and genetic methods has been found to attenuate inflammatory diseases, such as sepsis 25, lung inflammation 26, kidney inflammation 27, asthma 28, nonalcoholic steatohepatitis and fibrosis 29. Knockdown of lung MD‐2 by small interfering RNA in mice attenuates pollen‐ and cat dander‐induced innate and allergic airway inflammation 28. In kidney tissue, paclitaxel binds to MD‐2 to block MD‐2/TLR4 association during LPS treatment, resulting in the suppression of NF‐κB activation and inhibition of pro‐inflammatory cytokine production 27.

This research efforts exploring MD‐2‐targeting molecules to treat inflammatory diseases involve endogenous ligands, lipid‐like antagonists, natural and synthetic chemicals. KOdiA‐PC [1‐palmitoyl‐2‐(5‐keto‐6‐octenedioyl)‐*sn*‐glycero‐3‐phosphocholine], an oxidized phosphatidylcholine, inhibits the activation of TLR4 signalling pathway by suppressing the binding of LPS and MD‐2 30. More importantly, eritoran, a synthetic tetraacylated lipid A, antagonizes the binding of LPS to the same site of MD‐2. Eritoran showed exciting results in phases I and II clinical trials of severe sepsis 31, 32. However, eritoran failed its phase III clinical trial because there was no obvious improvement with the compound over placebo 33. Some natural and synthetic chemicals, such as curcumin 18, JSH 15, gedunin 34 and xanthohumol 17, and two new compounds, L6H21 24 and L48H37 19, which we investigated in our laboratory, bind directly to the MD‐2 hydrophobic pocket and showed obvious anti‐inflammatory activity in that they displace LPS from MD‐2. In this study, we found that L2H21 is an inhibitor of MD‐2 (Figs 1 and 2). In addition, L2H21 significantly reduces LPS‐induced inflammatory response both *in vitro* and *in vivo* (Figs 3 and 5). The described results further suggest that the role of MD‐2 in mediating inflammatory response and MD‐2 may serve as a good anti‐inflammatory target.

To explore the molecular mechanism of the L2H21 binding to MD‐2, molecular docking assay was used. Data from Figure 2A show that L2H21 binds with MD‐2 through Tyr^102^ and Arg^90^ residues. In previous reports, we identified that Tyr^102^ and Arg^90^ residues in the MD‐2 hydrophobic pocket play important roles in the interaction between MD‐2 protein and the chalcone analogue, L6H21 24. In fact, xanthohumaol, a prenylated chalcone‐type flavonoid, is positioned in the deep hydrophobic interior of the MD‐2 pocket when hydrogen bonded to the Tyr^102^ residue 17. Our results verify the importance of Try^102^ and Arg^90^ as molecular targets for MD‐2 inhibitors.

As induced by LPS, ALI is characterized by pulmonary oedema, alveolar–capillary barrier damage, cells recruitment and a cascade of inflammatory reactions 35. Thus far, no effective medications for ALI exist in clinical therapy, and the mortality rate remains very high. Recent studies report that MD‐2 inhibition attenuates lung inflammation. MD‐2‐null mice displayed no response to pulmonary inflammation after nasal aspiration of LPS from *Neisseria meningitides* 26. Tumurkhuu *et al*. report that the alternatively spliced short isoform of human MD‐2 inhibits Gram‐negative bacterial endotoxin‐induced lung inflammation 36. Here, the MD‐2 inhibitor, L2H21, markedly reduced LPS‐induced pulmonary oedema (Fig. 4C) and infiltration by neutrophils (Fig. 5B), which is the predominant feature of ALI. Meanwhile, L2H21 inhibited ALI‐induced inflammatory cytokines expression in BALF, serum and lung tissue (Fig. 5). In lung tissue, LPS induced an increase in TLR4/MD‐2 complex levels, whereas pre‐treatment with L2H21 inhibited that increase (Fig. 5N). Therefore, this translational study renders promise for the MD‐2 inhibitor, L2H21, as a candidate for the treatment of ALI, and verifies MD‐2 inhibition as a potential therapeutic strategy for ALI.

In conclusion, the various results demonstrate inhibition of MD‐2 by L2H21 and its ability to attenuate LPS‐induced ALI. These findings suggest that MD‐2 is an important therapeutic target against LPS‐induced ALI, and that its inhibitor, L2H21, is a promising treatment agent.

## Conflict of interest

The authors confirm that there are no conflicts of interest.
